# ﻿*Carcinoplaxmistio* Ng & Mitra, 2019 (Crustacea, Decapoda, Goneplacidae): additional records and genetic differentiation of allied taxa

**DOI:** 10.3897/zookeys.1214.131500

**Published:** 2024-10-01

**Authors:** Mani Prema, Chien-Hui Yang, Samuthirapandian Ravichandran, Peter K. L. Ng

**Affiliations:** 1 Centre of Advanced Study in Marine Biology, Faculty of Marine Sciences, Annamalai University, Parangipettai–608 502, India Annamalai University Parangipettai India; 2 Centre for Marine Living Resources and Ecology, Ministry of Earth Sciences, Kochi – 682508, Kerala, India Ministry of Earth Sciences Kochi India; 3 Institute of Marine Biology and Center of Excellence for the Oceans, National Taiwan Ocean University, 2 Pei-Ning Road, Keelung 202231, Taiwan National Taiwan Ocean University Keelung Taiwan; 4 Department of Zoology, Government Arts & Science College, Nagercoil – 629 004, India Government Arts & Science College Nagercoil India; 5 Lee Kong Chian Natural History Museum, Faculty of Science, National University of Singapore, 2 Conservatory Drive, Singapore 117377, Singapore National University of Singapore Singapore Singapore

**Keywords:** Brachyura, COI, genetic and morphological incongruence, goneplacid crab, Goneplacoidea, India, systematics, 16S rRNA

## Abstract

The goneplacid crab, *Carcinoplaxmistio* Ng & Mitra, 2019, was originally described from West Bengal, India, in the northern Indian Ocean. Additional material of *C.mistio* from off Tamil Nadu in the southeast of India revealed a high degree of size-associated variation in the structures of the anterolateral tooth of the carapace, chelipeds, and male and female pleons. In addition to an in-depth morphological examination of *C.mistio*, this study also records the natural coloration of the species and conducts a genetic comparison (with mitochondrial COI and 16S rRNA genes) with its close relatives, *C.haswelli* (Miers, 1884) and *C.purpurea* Rathbun, 1914. Molecular comparison of *C.mistio* with its morphologically closest congener, *C.haswelli* from northern Australia and the western Pacific, corroborates their status as separate species. The genetic sequence of *C.mistio*, however, is similar to that of *C.purpurea* from the West Pacific, although these two species can easily be distinguished by distinct carapace and ambulatory leg characters. The present study provides some possible explanations for the genetic and morphological incongruence observed between *C.mistio* and *C.purpurea* and highlights the need for a detailed molecular study for *Carcinoplax* H. Milne Edwards, 1852, to appreciate the evolution of various morphological characters in the genus.

## ﻿Introduction

The goneplacid crab genus, *Carcinopla*x H. Milne Edwards, 1852, comprises 26 species from the Indo-West Pacific (Castro 2007, 2009; Ng and Kumar 2016; Ng and Mitra 2019; Ng and Castro 2020). Six species of *Carcinopla*x are known from India: *C.longimanus* (De Haan, 1833); *C.longipes* (Wood-Mason, in Wood-Mason & Alcock, 1891); *C.indica* Doflein, 1904; *C.specularis* Rathbun, 1914; *C.fasciata* Ng & Kumar, 2016; and *C.mistio* Ng & Mitra, 2019 (see Trivedi et al. 2018; Ng and Mitra 2019).

While describing *C.mistio* from West Bengal, India, Ng and Mitra (2019) compared it with two close congeners, *C.sinica* Chen, 1984, and *C.purpurea* Rathbun, 1914, noting that *C.mistio* has a combination of diagnostic characters of other species. *Carcinoplaxsinica* has since been synonymised with *C.haswelli* (Miers, 1884) (cf. Ng et al. 2022). Ng and Mitra (2019) had only three specimens of *C.mistio* available for study, so they were unable to assess allometric variation, which can be pronounced in some species of *Carcinoplax* (Guinot 1989; Castro 2007). A good series of *C.mistio* was recently collected from Tamil Nadu, southeast coast of India, allowing the present evaluation of morphological variation in the species. The present study also takes the opportunity to document the natural coloration of *C.mistio* and compare the genetics of allied *C.mistio*, *C.haswelli* and *C.purpurea*.

## ﻿Material and methods

### ﻿Material

The material used for morphological examination is deposited in the Zoological Survey of India, Kolkata (**ZSIK**); and Centre of Advanced Study in Marine Biology, Annamalai University, Parangipettai, Tamil Nadu (**CASAU**). Details of all specimens examined are provided in the material examined subsection of the systematic account below. Measurements provided, in millimetres (**mm**), are of the maximum carapace width (inclusive of spines) and length (taken at the midline from the tips of the frontal margin to the median part of the posterior margin), respectively. The terminology used follows Davie et al. (2015) and Ng and Mitra (2019). Abbreviations used in this study are as follows: **coll.** = collector; **juv.** = juvenile; **G1** = male first gonopod; and **G2** = male second gonopod; **ovig.** = ovigerous.

### ﻿Molecular analysis

In addition to *C.mistio*, *C.haswelli* and *C.purpurea*, four other species of *Carcinoplax* [*C.ischurodous* (Stebbing, 1923), *C.longimanus*, *C.nana* Guinot, 1989 and *C.tomentosa* Sakai, 1969], are also included for the molecular analysis. *Goneplaxrhomboides* (Linnaeus, 1758) was selected as the outgroup. The samples for molecular analyses were from the Zoological Reference Collection of the Lee Kong Chian Natural History Museum, National University of Singapore (ZRC), and National Taiwan Ocean University (NTOU) (Table 1).

**Table 1.** Material, sampling localities and GenBank accession numbers of *Carcinoplax* and outgroup used in this study. “#” sequences downloaded from GenBank. N.C. - no sequence available.

Species	Locality	Voucher Nos.	GenBank Accession Nos.	
(code)			COI	16S rDNA
*C.haswelli* (1)	Gulf of Tonkin	ZRC 2011.0607	OP163291	PQ163823
*C.haswelli* (2)	Off Singapore	ZRC 1984.5693	OP163292	N.C.
*C.ischurodous*			MZ434779 ^#^	MZ424933 ^#^
*C.longimanus* (1)	Taiwan	NTOU B00138	OP163293	PQ163824
*C.longimanus* (2)			MZ434781 ^#^	MZ424935 ^#^
*C.longimanus* (3)			MZ434783 ^#^	MZ424936 ^#^
*C.mistio* (1)	India	ZRC 2022.0812 (male)	OP163294	PQ163825
*C.mistio* (2)	India	ZRC 2022.0812 (female)	OP163295	PQ163826
*C.nana*	Philippines	ZRC 2019.0361	OP163296	PQ163827
*C.purpurea* (1)	Taiwan	ZRC 2001.0017	OP163297	PQ163828
*C.purpurea* (2)	Taiwan	NTOU B00139	OP163298	PQ163829
*C.purpurea* (3)	Philippines	ZRC 2006.0216	OP163299	PQ163830
*C.tomentosa*	Taiwan	NTOU B00140	OP163300	PQ163831
*Goneplaxrhomboides*			MG935224 ^#^	JN591672 ^#^

Crude genomic DNA was extracted from the muscles of the pleon using a DNeasy® Blood and Tissue Kit (Qiagen, Hilden, Germany) following the protocol of the manufacturer. Molecular markers were selected as the mitochondrial COI and 16S rRNA genes, while the sequences amplification using LCO1490/HCO2198 (~657 bp, Folmer et al. 1994) and 16Sar/16S1472 (~550 bp) (Simon et al. 1994; Crandall and Fitzpatrick 1996), respectively. PCR reactions, cycling profiles, product checking and sequencing procedures followed those used in Ng et al. (2018). The output sequences were edited for contig assembly by SeqMan Pro^TM^ (Lasergene®; DNASTAR, Madison, WI, USA), then blasted on the GenBank (National Center for Biotechnology Information, NCBI) to check for any potential contamination. EditSeq (Lasergene®; DNASTAR) was used to translate into the corresponding amino acid sequences to avoid the inclusion of pseudogenes for the COI dataset (Song et al. 2008). Sequence alignment and nucleotide pairwise distance for each of the two datasets were calculated based on the Kimura 2-parameter model (K2P, Kimura 1980) by MEGA v.11 (Tamura et al. 2021). The maximum-likelihood (ML) tree was constructed based on the combined sequences (COI+16S rDNA) using MEGA v.11 by 1000 bootstrap replicates (Felsenstein 1985). We failed to get a complete 16S rDNA sequence on *C.haswelli* (ZRC 1984.5693), and the missing sequence was filled up by the fifth nucleotide “N” for the combined dataset.

## ﻿Systematic account

**Superfamily Goneplacoidea MacLeay, 1838**

**Family Goneplacidae MacLeay, 1838**

**Genus *Carcinoplax* H. Milne Edwards, 1852**

### ﻿ Carcinoplax mistio

Taxon classificationAnimaliaDecapodaGoneplacidae

Ng & Mitra, 2019

308DA7EC-D150-5B72-A3E1-F9A9BDB4C05C

Figs 1 , 2 , 3

**Figure 1.**
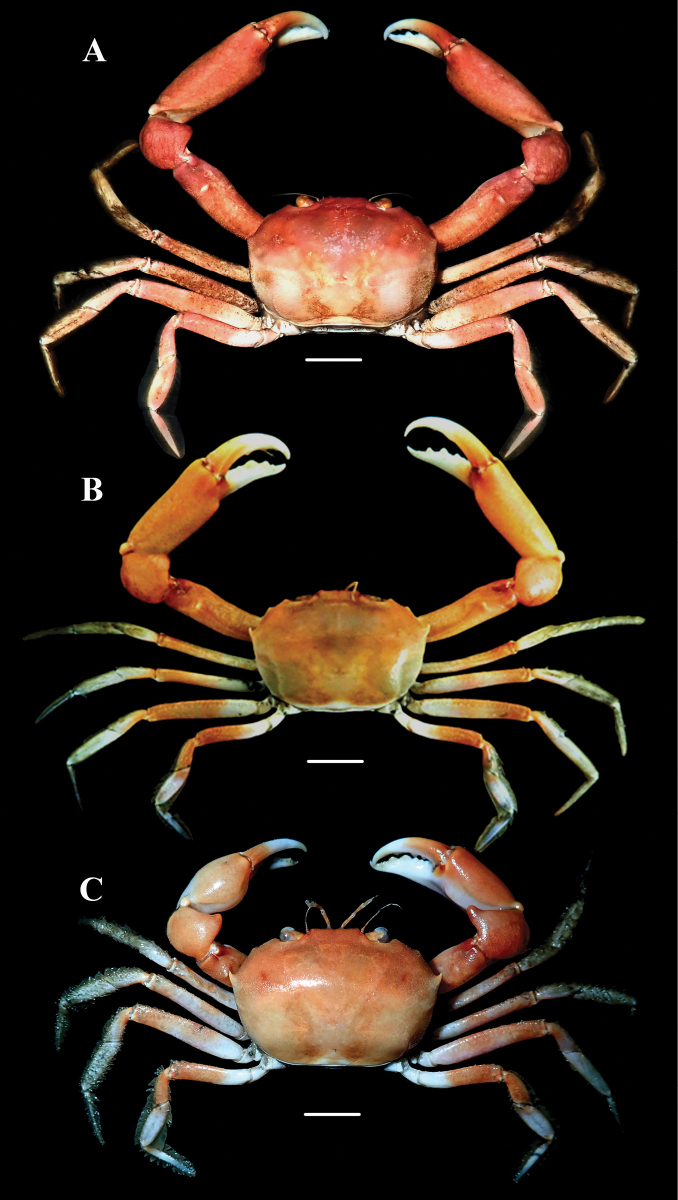
Colour in life and overall dorsal view of *Carcinoplaxmistio* Ng & Mitra, 2019 **A** male (33.3 × 23.4 mm) (CASAU CR-1037) **B** male (29.2 × 20.5 mm) (CASAU CR-1036) **C** female (33.5 × 24.0 mm) (CASAU CR-1040). Scale bars: 1.0 cm (**A–C**).

**Figure 2.**
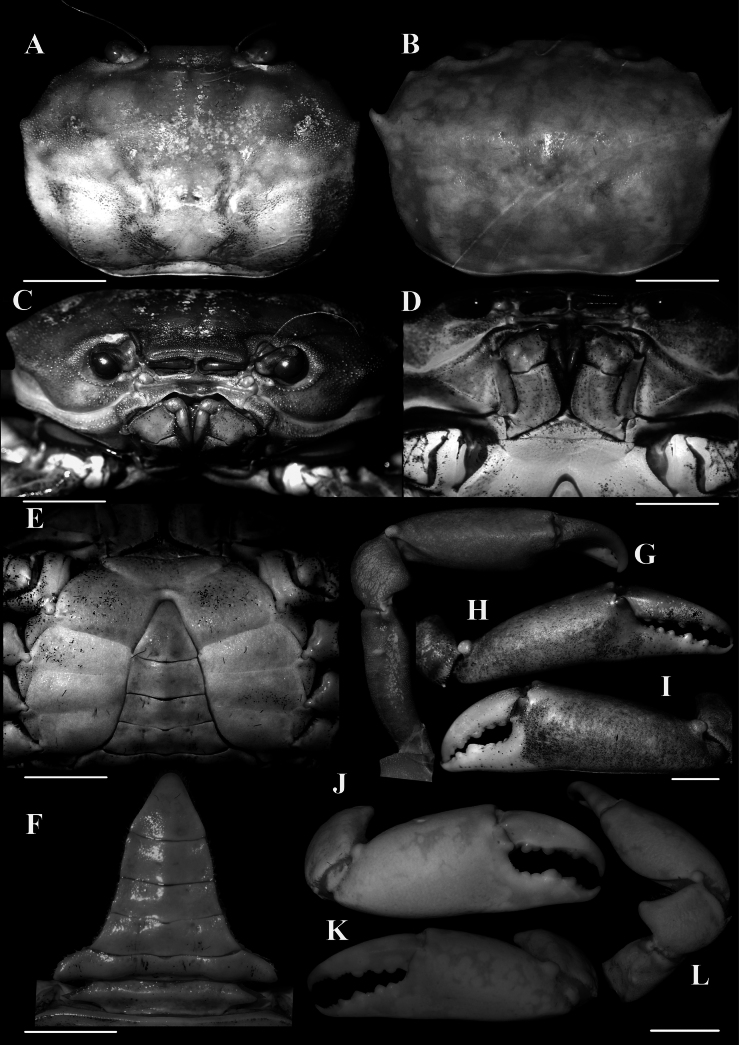
*Carcinoplaxmistio* Ng & Mitra, 2019 **A, C, D, E–I** male (33.3 × 23.4 mm) (CASAU CR-1037) **B, J–L** male (24.8 × 16.4 mm) (CASAU CR-1039) **A, B** dorsal view of carapace **C** frontal view of cephalothorax **D** third maxillipeds **E** thoracic sternites 3–6, pleonal somites and telson **E** pleonal somites and telson **G–L** dorsal and outer views of chelae. Scale bars: 5.0 mm (**A–L**).

Carcinoplax (purpurea) ? – Stephensen 1946: 166, 208, fig. 44 (not Carcinoplaxpurpurea Rathbun, 1914).
Carcinoplax purpurea – Guinot 1967: 276 (list); Titgen 1982: 252 (list) (not Carcinoplaxpurpurea Rathbun, 1914).
Carcinoplax sinica – Guinot 1989: 285, fig. 14A, B, pl. 5 figs A, B, B1, C, C1, D, E, E1; Apel 2001: 101; Naderloo and Sari 2007: 449; Naderloo 2017: 69, text-fig. 11.2d, e, fig. 12.1 (not Carcinoplaxsinica Chen, 1984) [= Carcinoplaxhaswelli Miers, 1884)].
Carcinoplax mistio Ng & Mitra, 2019: e2019004, figs 1, 2, 6A, 7A, G, H, 8A–G, 9A, B.
Carcinoplax haswelli – Sureandiran et al. 2024: figs 2–7 (not Carcinoplaxhaswelli Miers, 1884).

#### Material examined.

***Holotype*.** India • ♂ (29.2 × 19.0 mm); northern Bay of Bengal, Fresargunj Fishing Harbour; 24 Feb. 2017; coll. local fishermen by trawl; ZSIK C7123/2. **Paratypes.** India • 1 ♀ (36.4 × 24.2 mm); same collection data as for holotype; ZSIK C7124/2 • 1 ♀ (36.7 × 27.5 mm); northern Bay of Bengal, Fresargunj Fishing Harbour; 28 Jul. 2018; ZSIK.

#### Other material.

India • 4 ♂♂ (35.1 × 30.0 mm, 29.2 × 22.3 mm, 25.1 × 18.2 mm, 23.2 × 29.2 mm), 5 ♀♀ (37.1 × 31.0 mm, 36.2 × 30.1 mm, 32.1 × 25.2 mm, 31.0 × 25.2 mm, 26.1 × 24.0 mm); southern Bay of Bengal, eastern Tamil Nadu, Pazhayar Fishing Port; 11°21'N, 79°50'E; depth 50–100 m; 2016–2020; coll. M. Prema & S. Ravichandran; CASAU CR-1031 • 1 ♂ (29.7 × 19.6 mm), 1 ♀ (43.2 × 29.2 mm); same collection data as for preceding; 2016–2020; CASAU CR-1032 • 2 ♂♂ (37.6 × 25.8 mm, 32.0 × 21.3 mm), 7 ♀♀ (37.9 × 24.5 mm, 37.0 × 25.1 mm, 34.0 × 22.9 mm, 32.3 × 21.6 mm, 31.2 × 21.1 mm, 29.5 × 19.6 mm, 27.6 × 25.6 mm,); same collection data as for preceding; 18 Mar. 2018; CASAU CR-1033 • 1 ♂ (26.4 × 18.0 mm), 1 juv. ♀ (19.7 × 13.4 mm); same collection data as for preceding; Mar. 2018; CASAU CR-1034 • 4 ♂♂ (36.0 × 23.8 mm, 33.0 × 22.7 mm, 32.5 × 22.2 mm, 31.5 × 21.4 mm), 1 ovig. ♀ (39.0 × 26.6 mm), 1 ♀ (36.1 × 23.4 mm); same collection data as for preceding; 2020–2021; CASAU CR-1035 • 3 ♂♂ (29.2 × 20.8 mm, 29.2 × 20.5 mm, 27.6 × 19.4 mm); same collection data as for preceding; 12 Jan. 2022; CASAU CR-1036 • 1 ♂ (33.× 23.4 mm); same collection data as for preceding; CASAU CR-1037 • 2 ♀♀ (37.3 × 24.6 mm, 35.9 × 23.7 mm); same collection data as for preceding; 26 Mar. 2023; CASAU CR-1038 • 2 ♂♂ (26.2 × 18.1 mm, 24.8 × 16.4 mm), 2 ♀♀ (32.2 × 22.0 mm, 29.5 × 19.4 mm); same collection data as for preceding; 11 Feb. 2024; CASAU CR-1039 • 7 ♀♀ (37.9 × 26.0 mm, 36.2 × 24.3 mm, 34.7 × 22.5 mm, 33.5 × 24.0 mm, 30.5 × 20.5 mm, 29.2 × 20.1 mm, 25.1 × 17.1 mm); same collection data as for preceding; 11 Feb. 2024; CASAU CR-1040.

#### Diagnosis.

**Figure 3.**
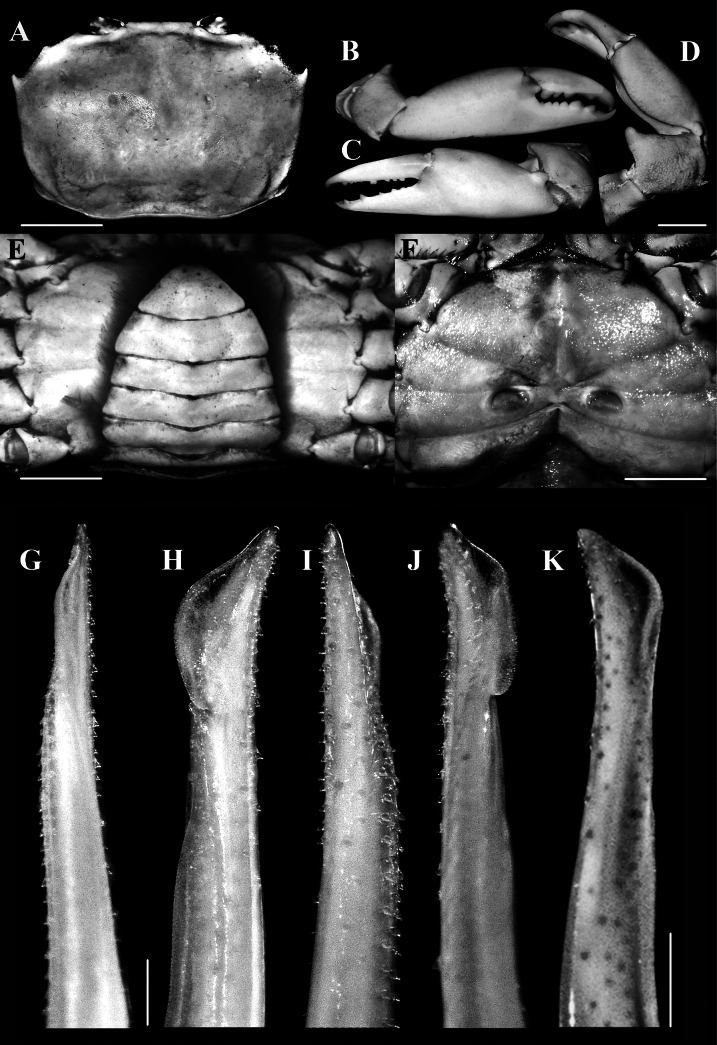
*Carcinoplaxmistio* Ng & Mitra, 2019 **A–F** female (33.5 × 24.0 mm) (CASAU CR-1040); **G– J** male (33.3 × 23.4 mm) (CASAU CR-1037) **K** male (25.1 × 18.2 mm); **A** dorsal view of carapace **B–D** dorsal and outer views of chelae **E** pleon and telson **F** thoracic sternites with position of vulvae **G** dorsal view of left G1**H** dorso-lateral view of left G1**I** ventral view of left G1**J** ventro-lateral view of left G1**K** lateral view of left G1. Scale bars: 5.0 mm (**A–F**); 1.0 mm (**G–K**).

Modified from Ng and Mitra (2019). Carapace broad, dorsal surface gently convex; antero-lateral surfaces generally with small, rounded, densely packed granules, sometimes appearing almost smooth; post-orbital region with small, rounded granules; second anterolateral teeth relatively short in larger specimens, slightly sharp in smaller specimens; gastro-cardiac groove shallow but visible (Figs 1, 2A, B, 3A). Chelipeds unequal, male carpal spine more rounded, that on female more elongate (Figs 1, 2G–I, J–L, 3B–D). Ambulatory legs long, slender; articles laterally flattened, smooth; margins lined with setae (Fig. 1). G1 relatively slender, laterally flattened, tip elongate, tapering, lined with numerous short spines (Fig. 3G–K). G2 longer than G1, distal segment long, curved, tip weakly bifurcated (Ng and Mitra 2019: fig. 8D).

#### Habitat.

The present specimens of *C.mistio* were collected from 50–100 m depth, off the coastal waters of Tamil Nadu state, Bay of Bengal, India. The three type specimens were obtained from West Bengal, also from a fishing port but without depth data (Ng and Mitra 2019).

#### Coloration in life.

Carapace orange, cheliped fingers and upper surface of ambulatory legs white (Fig. 1A–C), merus of ambulatory leg generally orange (Fig. 1), and sternopleonal surfaces pale white.

#### Distribution.

Northern Indian Ocean: Bay of Bengal (West Bengal and off Tamil Nadu coast, India; Ng and Mitra 2019; present study); north-western Arabian Sea (Gujarat, India; Sureandiran et al. 2024); and Persian Gulf (Guinot 1989; Naderloo 2017).

#### Remarks.

The present specimens of *C.mistio* agree well with the type account (Ng and Mitra 2019). The large series of specimens, however, allowed us to document size-related morphological variation. The largest specimens of *C.mistio* collected in this study have a carapace width of 37.6 mm (male, CASAU CR-1033) and 43.2 mm (female, CASAU CR-1032), respectively; both are larger than the type specimens and are the largest known specimens of the species.

In the types as well as in the smaller males (e.g., 26.4 × 18.0 mm, CASAU CR-1034; 24.8 × 16.4 mm, CASAU CR-1039) and most of the larger specimens of the present collection, the second anterolateral tooth of the carapace is prominent, being sharp and curved (Figs 1B, 2B). In the largest males (e.g., 35.1 × 30.0 mm, CASAU CR-1031; 33.3 × 23.4 mm, CASAU CR-1037), this tooth is relatively lower (Figs 1A, 2A) and comparable to the condition in *C.purpurea*. In *C.purpurea*, however, the second anterolateral tooth is even lower and more like a rounded tubercle (cf. Ng and Mitra 2019: fig. 6C, D). As such, the form of the second anterolateral tooth is not a reliable diagnostic character for *C.mistio* at all body sizes, being sometimes size dependent, though it is usually sharp and longer. The cheliped of the largest males is elongate, with the merus and fingers extremely long (Fig. 1A, B, 2G–I), a condition like that of *C.longimanus* (see Guinot 1989). In the smaller holotype male of *C.mistio* as well as in smaller males, the chelipeds are relatively shorter (Fig. 2J–L). Sexual dimorphism is apparent as all females have relatively shorter cheliped fingers (Figs 1C, 3B–D).

The cheliped carpal spine of male *C.mistio* specimens examined, including the holotype male, is relatively more rounded and relatively shorter (Figs 1A, B, 2G, H, J, L) (versus the cheliped carpal spine being relatively less rounded, more elongate and curved in most of the females; Figs 1C, 3B, D). In the holotype male of *C.mistio*, the carpal spine is relatively short and rounded (Ng and Mitra 2019: fig. 1A, F) and as such, its length is a sexually dimorphic character (Ng and Mitra 2019: fig. 2A, D, F) that is not size dependent. This is similar to the condition of the cheliped carpal spine that was reported for *C.haswelli* (as *C.sinica*, cf. Ng and Mitra 2019: fig. 4E).

The lateral margins of pleonal somite 6 of large males is gently convex, gradually converging towards the telson, which is similar to that of the holotype of *C.mistio* (Fig. 2E, F; see Ng and Mitra 2019: fig. 7A). In the large male of *C.mistio* (33.3 × 23.4 mm, CASAU CR-1037), pleonal somite 6 is proportionately broader, width-to-length ratios 2.1 (versus pleonal somite 6 width-to-length ratios in two smaller males (26.2 × 18.1 mm, 24.8 × 16.4 mm, CASAU CR-1040) being 1.96 and 1.98, respectively). The pleon of large females in the present collection is similar to that reported for the paratype *C.mistio* (36.4 × 24.2 mm, ZSIK C7124/2) (cf. Ng and Mitra 2019: fig. 9A), but in a smaller specimen (26.1 × 24.0 mm, CASAU CR-1031), the pleon is relatively wider than in the paratype. Among the 28 female specimens studied, only one was ovigerous (39.0 × 26.6 mm, CASAU CR-1035). In juvenile females (e.g., 19.7 × 13.4 mm, CASAU CR-1034), the pleon is not expanded, lacking setae on pleopods, and the operculum of the vulva is poorly developed.

The proportions of the male telson vary regardless of size with the width-to-length ratios of three males (33.3 × 23.4 mm, CASAU CR-1037; 26.2 × 18.1 mm, 24.8 × 16.4 mm, CASAU CR-1040) are 0.76, 0.88 and 0.67, respectively. Overall, the male telson is slightly broader with the lateral margins being more concave (Fig. 2E, F) than in *C.haswelli* (cf. Ng and Mitra 2019: fig. 7D–F).

The mesial margin of the distal two-thirds of the G1 is gently concave in large specimens of *C.mistio* (Fig. 3G) and almost straight in smaller ones (Fig. 3I), but the tip is always elongate and tapering (Fig. 3G–J). Ng and Mitra (2019) observed that the G1s of the holotype (29.2 × 19.0 mm, ZSIK C7123/2) were distally damaged. Sureandiran et al. (2024) reported “*Carcinoplaxhaswelli*” based on one male specimen from Gujarat in western India, but all their figures of the G1 and the carapace (see Sureandiran et al. 2024: figs 2–7), actually correspond to *C.mistio*.

**Figure 4.**
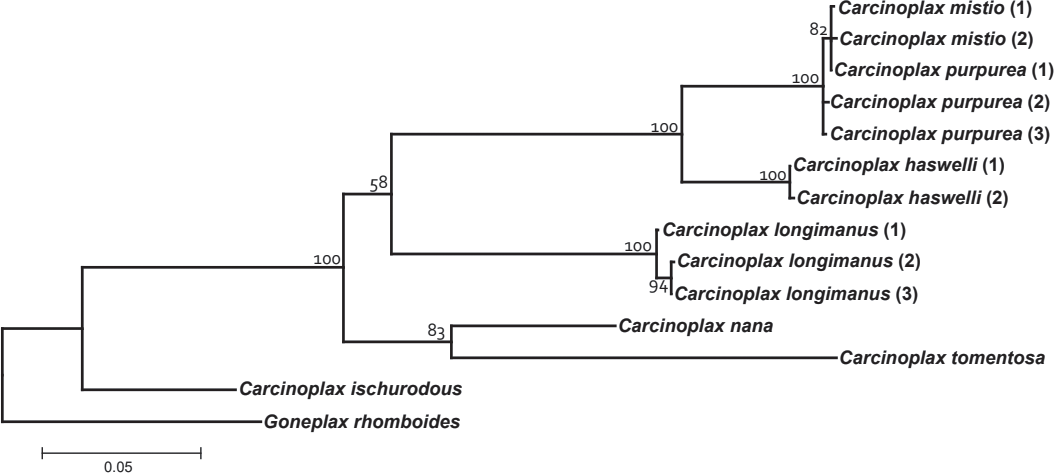
Maximum likelihood phylogenetic tree for seven species of *Carcinoplax* based on the mitochondrial COI+16S rRNA genes dataset. *Goneplaxrhomboides* (Linnaeus, 1758) was chosen as outgroup. Bootstrap value is represented above the branches. Values < 50 are not shown.

The genetic comparisons for seven species of *Carcinoplax*, including *C.mistio*, are interesting (Fig. 4). The intraspecific divergences of COI (657 bp) and 16S rRNA (552 bp) genes for four morphologically distinct species of *Carcinoplax* are less than 1.5%: *C.haswelli* (COI 0.2%), *C.mistio* (COI 0%, 16S 0.2%), *C.purpurea* (COI 0.5–1.1%, 16S 0%), and *C.longimanus* (COI 0.2–0.8%, 16S 0.0–0.4%) (Table 2). As for the interspecific divergences of the three species under study here (Table 2), that between *C.haswelli* and *C.mistio* is high (COI 10.3–10.5%, 16S 3.5–3.7%), as is that between *C.haswelli* and *C.purpurea* (COI 9.9–10.5%, 16S 3.5%) (Table 2, Fig. 4), corroborating their status as separate species. The genetic divergence between *C.mistio* and *C.purpurea*, however, was unexpectedly low (COI 0.3–0.8%, 16S 0.0–0.2%) and within the range normally considered for conspecificity (Fig. 4) when compared with the other four species of *Carcinoplax* (COI 12.4–21.1%, 16S 6.5–12.1%) (Table 2, Fig. 4). The morphological differences between *C.mistio* and *C.purpurea*, however, are substantial. In *C.mistio*, the carapace is proportionally wider, appearing more rectangular in shape with the posterolateral margins distinctly converging posteriorly (Figs 1A–C, 2A, B, 3A; see Ng and Mitra 2019: figs 1A, 2A, 6A, B) (versus carapace more quadrate with the posterolateral margins subparallel in *C.purpurea*; see Ng and Mitra 2019: figs 3A, 6C, D); the second (last) anterolateral tooth is usually sharp and curved (Figs 1B, C, 2B, 3A) (versus low and rounded in *C.purpurea*; see Ng and Mitra 2019: figs 3A, 6C, D); and the ambulatory legs are long and slender (Fig. 1A–C; see Ng and Mitra 2019: figs 1A, 2A, 7G, H) (versus distinctly shorter and stouter in *C.purpurea*; see Ng and Mitra 2019: figs 3A, 7I, J). Noteworthy is that the G1s of *C.mistio* and *C.purpurea* are similar (Fig. 3G–J; see Ng and Mitra 2019: fig. 8E, F, H, I). The characters of the G1 are more conservative in goneplacid evolution than carapace and pereopod differences, which are more plastic. Significant morphological differentiation but with low genetic variation has previously been reported in *Armasesangustipes* (Dana, 1852) (Sesarmidae, Marochi et al. 2017), *Carcinusmaenas* (Linnaeus, 1758) (Carcinidae, Silva et al. 2010), and *Pachygrapsusmarmoratus* (Fabricius, 1787) (Grapsidae, Deli et al. 2015). There are many possible explanations for this observed discordance, ranging from incomplete lineage sorting to retention of ancestral genotypes, etc. (see Meier et al. 2006; Tang et al. 2012; Nabholz 2023).

**Table 2.** Pairwise distance based on Kimura-2-parameter (K2P) model of partial mitochondrial COI (within and under the diagonal) and 16S rDNA (value in the brackets and above the diagonal) sequences among *Carcinoplax* species. *Goneplaxrhomboides* (Linnaeus, 1758) was treated as an outgroup.

	*Carcinoplaxhaswelli*	*C.ischurodous*	*C.longimanus*	*C.mistio*	*C.nana*	*C.purpurea*	*C.tomentosa*	*Goneplaxrhomboides*
*Carcinoplaxhaswelli*	0.002	0.102	0.098–0.101	0.035–0.037	0.105	0.035	0.134	0.128
*C.ischurodous*	0.199–0.202		0.078–0.083	0.095–0.097	0.080	0.095	0.093	0.071
*C.longimanus*	0.172–0.178	0.176–0.178	0.002–0.008 [0.0–0.004]	0.082–0.088	0.078–0.084	0.082–0.086	0.084–0.087	0.113
*C.mistio*	0.103–0.105	0.190	0.159–0.164	0.0 [0.002]	0.093–0.095	0.0–0.002	0.118–0.121	0.113–0.116
*C.nana*	0.165–0.167	0.154	0.124–0.127	0.179		0.093	0.065	0.105
*C.purpurea*	0.099–0.105	0.192–0.195	0.159–0.169	0.003–0.008	0.181–0.184	0.005–0.011 [0.0]	0.118	0.113
*C.tomentosa*	0.173–0.175	0.209	0.203–0.211	0.203	0.160	0.200–0.203		0.105
*Goneplaxrhomboides*	0.187–0.189	0.122	0.204–0.205	0.198	0.172	0.195–0.203	0.219	

A detailed molecular study of *Carcinoplax* will be necessary to appreciate the evolution of the various morphological characters in the genus as currently defined (sensu Castro 2007). *Carcinoplax* currently contains 26 species, all from the Indo-West Pacific, with many species morphologically similar and often occurring sympatrically, although several species span both oceans (see Castro 2007, 2009; Ng and Castro 2020). As the present study indicates, genetic and morphological incongruence may be more common in *Carcinoplax* than expected, and wide-ranging taxa may well prove to be species-complexes (see Ng and Castro 2020). Currently, *C.mistio* is known from the northern Indian Ocean, ranging from the Bay of Bengal to the Persian Gulf. *Carcinoplaxpurpurea* is only known for certain from the western Pacific (Castro 2007). There is also a record of *C.purpurea* from Madagascar by Castro (2007: 639), but it was based on a badly preserved male specimen, and it more likely belongs to either *C.monodi* Guinot, 1989, or *C.haswelli*. *Carcinoplaxhaswelli*, however, occurs in the western Pacific, Southeast Asia and eastern Indian Ocean (north-western Australia) (Ng et al. 2022).

## Supplementary Material

XML Treatment for Carcinoplax mistio

## ﻿Additional information

### Author ORCIDs

Mani Prema https://orcid.org/0000-0003-2694-3034

Chien-Hui Yang https://orcid.org/0000-0002-4594-3622

Samuthirapandian Ravichandran https://orcid.org/0000-0002-8632-5062

Peter K. L. Ng https://orcid.org/0000-0001-5946-0608

### Data availability

All of the data that support the findings of this study are available in the main text.
